# Canine Geriatric Syndrome: A Framework for Advancing Research in Veterinary Geroscience

**DOI:** 10.3389/fvets.2022.853743

**Published:** 2022-04-21

**Authors:** Brennen A. McKenzie, Frances L. Chen, Margaret E. Gruen, Natasha J. Olby

**Affiliations:** ^1^Cellular Longevity Inc., dba Loyal, San Francisco, CA, United States; ^2^College of Veterinary Medicine, North Carolina State University, Raleigh, NC, United States

**Keywords:** aging, lifespan, healthspan, quality of life, caregiver burden

## Abstract

Biological aging is the single most important risk factor for disease, disability, and ultimately death in geriatric dogs. The effects of aging in companion dogs also impose significant financial and psychological burdens on their human caregivers. The underlying physiologic processes of canine aging may be occult, or early signs of aging may be ignored because of the misconception that biological aging is natural and therefore inevitable. The ability to detect, quantify, and mitigate the deleterious processes of canine aging would greatly enhance veterinary preventative medicine and animal welfare. In this paper we propose a new conceptual framework for aging in dogs, the Canine Geriatric Syndrome (CGS). CGS consists of the multiple, interrelated physical, functional, behavioral, and metabolic changes that characterize canine aging as well as the resulting clinical manifestations, including frailty, diminished quality of life, and age-associated disease. We also identify potential key components of a CGS assessment tool, a clinical instrument that would enable veterinarians to diagnose CGS and would facilitate the development and testing of interventions to prolong healthspan and lifespan in dogs by directly targeting the biological mechanisms of aging. There are many gaps in our knowledge of the mechanisms and phenotype of aging in dogs that must be bridged before a CGS assessment tool can be deployed. The conceptual framework of CGS should facilitate identifying these gaps and should stimulate research to better characterize the processes and effects of aging in dogs and to identify the most promising preventative strategies to target these.

## Introduction

After achieving physical and sexual maturity, all dogs experience aging, which can be pragmatically defined as “the progressive accumulation of changes with time associated with or responsible for the ever-increasing susceptibility to disease and death” (1). Aging is the single most important risk factor for a wide variety of superficially unrelated diseases, including neoplasia, osteoarthritis, cardiac disease, neurodegenerative disease, and many others (1, 2). Aging-associated disease severely impacts animal welfare and creates a significant emotional and financial burden for the human caregivers of geriatric dogs (3, 4). Mitigating the impact of aging on morbidity, mortality, and quality of life would address a critical unmet need in veterinary preventative medicine.

The dog is also a particularly appropriate translational model in which to study aging and the interventions intended to prolong lifespan and healthspan (the period of time before the occurrence of significant age-associated disease or disability). Table 1 provides a glossary of geroscience terms used in this article. Unlike traditional laboratory models of aging, such as worms and rodents, companion dogs share living environments and many features of their lifecycle and aging phenotype with humans. They exhibit patterns of age-associated morbidity and mortality that are much more similar to those of humans than other species. However, they also age much more rapidly than humans, making observational and interventional studies of lifespan much more feasible (16–18).

**Table 1:** Glossary of key terms.

**Term**	**Definition**	**References**
Aging	“The progressive accumulation of changes with time associated with or responsible for the ever-increasing susceptibility to disease and death”	Harman (1)
Biological Age	The extent to which aging has impacted the robustness, resilience, and state of health and function in an individual as measured by physical, functional, and biomarker assessment (contrasted with chronological age-the period of time the individual has been alive)	Karasik et al. (5), Jylhävä et al. (6), Diebel and Rockwood (7)
Canine Geriatric Syndrome (CGS)	The constellation of physical, functional, and metabolic changes that characterize aging in dogs and predispose to age-related dysfunction and disease	
Frailty	An aging-associated loss of robustness, resilience, and function accompanied by an increased risk of disease, disability, and death	Hoogendijk (8), Walston (9)
Geroscience	The interdisciplinary domain exploring the mechanisms of aging and the links between these mechanisms and negative health outcomes.	Kennedy et al. (10), Sieera (11), Kaeberlein (12)
Healthspan	The period of time free from significant age-related disease or disability (contrasted with lifespan-the period of time alive)	Rowe (13), Kaeberlein (14)
Resilience	The ability of an organism to return to a state of baseline or optimal physiologic function following perturbations caused by external stressors.	Ukraintseva (15)
Robustness	The ability of an organism to maintain a state of baseline or optimal physiologic function in the face of external stressors.	Ukraintseva (15)

As a species, the dog has exceptional phenotypic and genotypic diversity. This is the result of intensive artificial selection, which has created breeds with lower genetic diversity within each breed and greater differences between breeds than can usually be seen in human populations. This facilitates investigation of genetic variables relevant to differences in lifespan and age-related health outcomes. The extensive annotation of the canine genome further supports such investigation (19).

Companion dogs also typically share the living environment of their human caregivers, including many potential environmental variables that may influence aging and health. Because of their popularity as pets, dogs participate in a large healthcare infrastructure similar to the human healthcare system. This facilitates the collection of extensive data on health risk factors and outcomes in dogs, supporting real-world clinical research on aging mechanisms and interventions. Canine biology and the role of dogs in human society make dogs an excellent translational model for human aging (16–19).

Aging in the dog, as in humans, leads to a loss of robustness (the ability to maintain an optimal state of physiologic functioning) and resilience (the ability to return to this state after perturbations caused by external stressors) (20). Age is traditionally quantified in terms of the passage of time. However, the length of time an organism has been alive (its chronological age) is not the most precise or useful measure of the impact aging has had on an individual's robustness, resilience, or health (its biological age). The two are correlated, but just as species age at different rates, so do individuals within a species. The biological age of a 10-year-old human is much lower than that of a 10-year-old dog even though their chronological age is the same. There are similar, though less dramatic, differences in biological age within a species. Veterinary geroscience currently lacks reliable tools to assess the biological age of individual animals.

A conceptual framework for evaluating age-related changes and a validated clinical assessment tool would allow veterinarians to more effectively assess and manage aging as a risk factor for disease and mortality in individual patients. The purpose of this article is to propose and describe such a framework and to outline potential core components of such a clinical instrument.

## Canine Geriatric Syndrome

We propose the framework of Canine Geriatric Syndrome (CGS) to describe the constellation of key physical, functional, and metabolic changes that characterize aging in dogs and predispose to age-related dysfunction, disease, and death. Diagnosis of CGS would be made using a clinical assessment tool incorporating evaluation of physical function, behavioral and metabolic health, age-associated disease occurrence, frailty, quality of life, and caregiver burden. The components of this tool would include owner and veterinarian assessments, elements from the medical history, physical examination, and routine clinical laboratory testing as well as the use of specific diagnostic instruments to measure age-related change, such as tools for measuring frailty and cognitive function.

Though it is currently impossible to precisely predict the diseases or dysfunction that will result from aging in any individual dog, there are consistent patterns in the manifestations of canine aging. A clearer understanding of the components of the canine aging phenotype would improve our ability to measure biological age and predict health outcomes.

Similarly, there is a complex but limited set of physiologic pathways associated with aging and responsible for its detrimental effects on health and quality of life. Decades of research in multiple species have elucidated many of these pathways and shown them to be very similar across taxa (21–23). Contrary to the common view that aging is inscrutable and inevitable, this research shows that aging is simply another aspect of animal biology, intricate and variable yet comprehensible and amenable to intervention.

While the visible manifestations of aging are readily recognized, the underlying mechanisms often operate for many years before significant changes in appearance and function are detectable. Veterinarians and dog owners are unlikely to be aware of the signs of aging until a relatively late stage. We often dismiss early signs of age-related deterioration as insignificant or “normal,” and therefore not something to assess or treat. The view of aging as a natural and inevitable phenomenon hinders our ability to recognize, monitor, and delay its progression in the clinical setting and to preserve health and function in our aging canine patients.

### Existing Risk Assessment & Prediction Tools

Use of the CGS framework and associated assessment tool would facilitate earlier identification of individuals at risk for multiple age-related morbidities and mortality, and this assessment would trigger monitoring and intervention to reduce these risks. Such preemptive risk assessment and intervention is already part of veterinary practice, though it is applied to detecting and staging specific diseases rather than as a holistic assessment of aging as a risk factor for multi-morbidity.

A familiar example is the assessment and management of myxomatous mitral valve disease (MMVD). For many years, veterinarians could make a presumptive diagnosis of MMVD based on signalment and physical exam findings. However, without an ability to grade the severity of disease, and without therapeutic interventions shown to delay progression, clinicians were forced to wait until the late-stage onset of congestive heart failure before initiating treatment.

The development of the American College of Veterinary Internal Medicine (ACVIM) staging framework for MMVD (24), and the validation of pimobendan as an effective intervention to delay the onset of CHF in dogs with preclinical MMVD (25), have allowed veterinarians to more effectively identify and mitigate the risk of clinical disease preemptively in dogs with this condition. The CGS framework and clinical assessment could potentially serve a similar function with a much broader scope, staging the impacts of biological aging and facilitating prediction and prevention of the multiple health problems aging causes.

There are conceptual models and associated diagnostic instruments similar to our proposed CGS already in use in human medicine. One of these is Metabolic Syndrome (MS), a set of measurable risk factors for a variety of specific diseases that is used to assess metabolic health and guide preventative clinical interventions (26). The specific components of MS include: visceral obesity, impaired glucose metabolism, dyslipidemia, and hypertension. The syndrome is commonly diagnosed as the presence of three or more of these risk factors (27).

These components of MS are all related to aging, with most occurring more commonly with age across many different human populations (26, 28–30). The elements of MS include both measurable physical exam findings (e.g., waist circumference) and clinical laboratory measurements (e.g., fasting blood glucose). Some components can be defined as clinical disorders in themselves (e.g., hypertension), while others are not considered diseases *per se*. However, the purpose of assessing these components together is that they represent age-related dysfunction that can signal an increased risk of various clinical disorders, such as heart attacks and stroke, diabetes mellitus, and possibly chronic inflammatory diseases such as rheumatoid arthritis (31) and chronic obstructive pulmonary disease (32).

A CGS diagnostic tool would function similarly to MS, allowing a general assessment of the impact of age-related change on the overall morbidity and mortality risk for canine patients. While some clinical manifestations of aging differ between humans and dogs, many of the core mechanisms operate similarly for both species, and a composite risk assessment strategy would be useful for dogs as it has proven to be for humans.

### Identifying Knowledge Gaps

In order to operationalize the CGS framework and diagnostic instrument, there are many knowledge gaps that must be bridged between the fundamental biology of mammalian aging and the clinical prediction of morbidity and mortality risk in individual dogs. One objective of this paper is to identify areas in which further research is needed to fully develop and refine the CGS concept. Another goal is to identify those knowledge gaps that must be filled in order to design the CGS assessment tool and validate its clinical utility. These gaps represent opportunities for veterinary researchers to advance our understanding of canine aging and support the development of clinical interventions to prolong lifespan and healthspan in dogs.

Research is needed to select specific components of the CGS assessment tool and validate their utility in determining biological age and predicting morbidity and mortality. Some potential components are described below, along with areas for further investigation. Studies linking recognized biological mechanisms of aging to specific clinical manifestations in dogs are also crucial for identifying relevant biomarkers and therapeutic targets. Finally, beyond the biology of aging, a better understanding of the animal welfare consequences of age-related disease in dogs, the extent of the burden these places on human caregivers, and the financial costs of managing age-associated disease in dogs will be invaluable in targeting research efforts and resources most effectively to reduce the negative impacts of aging and age-associated disease.

### Potential Components of a CGS Assessment Tool

In addition to the value of CGS in reframing aging as a modifiable risk factor influencing variation in quality of life, frailty, and age-related disease incidence, the ultimate goal is to develop a clinical assessment tool for CGS. The practical implementation of this assessment will require selecting elements of the canine aging phenotype and underlying aging mechanisms that clinicians can readily identify and measure. These elements are likely to come from several broad domains of age-related changes in dogs:

- Physical changes
- Functional changes
- Behavioral changes
- Metabolic changes and biomarker candidates
- Frailty
- Quality of life and caregiver burden
- Age-associated clinical disease

#### Physical Changes

The physical aspects of the canine aging phenotype have been described previously (33). Some of these changes have been shown to be predictive of chronological age, such as the opacification of the lens (34). Others have been shown to be predictive of disease and mortality risk, such as body condition score (35). Still other measurable clinical features of aging in dogs have also been identified in humans as markers of increased risk for disease and death, including visceral adiposity (36) and sarcopenia (37). Finally, there are readily identifiable signs of aging that may or may not correlate with health outcomes, such as graying hair (38, 39).

Including characteristic phenotypic markers of canine aging that can be easily identified in practice and shown to owners during the CGS assessment as evidence of age-related health risk would support more effective preventative care for aging dogs. There have been some attempts to use specific clinical manifestations of aging to predict chronological age, such as lens opacity (40), graying of facial hair (41), and the extent of dental disease (42, 43). However, to date these physical manifestations of aging have only been shown to correlate with chronological age. The goal of the CGS assessment is to measure biological age and identify age-related morbidity and mortality risk. An important gap to be filled in our knowledge of the relationship between physical manifestations of aging and health outcomes is how predictive specific changes are of disease and mortality.

There is some evidence in humans, for example, that common physical manifestations of aging, such as skin wrinkles, hair graying, and even perceived age as rated by others, can predict mortality risk, but the findings are inconsistent (38, 39, 44). Further research in dogs is necessary to clarify the degree to which inclusion of visible physical manifestations of aging would strengthen the clinical utility of the CGS assessment.

#### Functional Changes

Many aspects of physical and behavioral function are key markers of aging and predictors of mortality in humans. Instruments testing physical function, such as the Short Physical Performance Battery (SPPB), and more general assessments of activities of daily living (ADL) are commonly used to assess functional status in humans, and these have been shown to be predictive of mortality (45–47).

Anecdotally, veterinarians and dog owners recognize functional decline in aging dogs, including lower levels of activity, slower walking speed, and difficulty with specific activities, such as climbing stairs or walking on slick surfaces. There are also studies specifically assessing changes in activity with age in dogs. They generally show reductions in spontaneous activity and functional capacity, but the findings are mixed and vary with breed, context, health, and other factors (48–54).

An important functional decline that deserves greater attention is the loss of continence, both fecal and urinary. The relative contributions of behavioral changes, loss of mobility, and decline in the functional competency of the neuromuscular structures involved have not been examined. The implications of this decline for caregiver burden, and the association between incontinence and the risk of euthanasia, is an area ripe for further investigation.

The potential associations between specific activity parameters and lifespan or mortality risk have not been examined in dogs. Given the strong established connection between functional capacity and mortality in humans, this is a critical subject for further investigation in dogs as part of the larger project to develop and operationalize the CGS assessment. Establishing the predictive value of specific changes in activity level would provide a powerful tool for early intervention to extend physical function and lifespan.

#### Behavioral Changes

Behavioral function also changes significantly with age, in humans and in dogs. There are many challenges in assessing these changes and in delineating the often indistinct boundaries between general aging of the brain, discrete or formally diagnosed neurologic disease, and the behavioral manifestations of aging changes in the rest of the body. For example, an aged dog may be less active due to diminished drive, loss of muscle strength, or musculoskeletal pain, and the relative role of these often concurrent age-related changes can be challenging to disentangle.

Age-associated changes in behavior occur on a continuum, and there is much debate about how we should distinguish normal aging from pathology (55, 56). When behavioral changes in older dogs are attributed to normal aging, veterinarians and owners tend not to pursue further assessment or intervention. Once a formal diagnosis is made, such as Canine Cognitive Dysfunction Syndrome (CDS), then treatment is more likely to be pursued. However, limitations in diagnostic tools for CDS may preclude diagnosis, and treatment options are also limited. Moreover, as in humans, there are likely many potential causes for progressive dementia in dogs, but the clinical, imaging, and histopathological characteristics of conditions other than CDS are poorly described at this time, again limiting our ability to recognize and appropriately treat dementia in dogs.

One purpose of the CGS framework is to encourage the recognition that aging and age-associated changes which diminish function and quality of life should be viewed as targets for prevention and mitigation. Assessing the extent of age-related behavioral change and incorporating this into a global measure of the impact of age on function and resilience will facilitate preventative and palliative measures to limit the effects of aging on both physical and behavioral capacity.

Numerous instruments are commonly used to assess age-associated changes in cognition, mood, and other behavioral domains in humans. Deficits identified by these are often clearly associated with increased mortality risk and diminished quality of life. This holds true even when the impairment does not meet criteria for diagnosis of specific disorders, such as dementia, Alzheimer's disease, or major depression (57–59).

Most instruments used in dogs to assess age-associated behavior change are intended to establish a diagnosis of CDS. These typically ask owners to rate the frequency of behavioral changes related to disorientation, changes in sleep-wake cycle (particularly sleeping during the day and being up during the night), changes in social interactions, new anxiety, and new house soiling. Progression of signs may be most observed in disorientation and social domains (60). In addition to signs of CDS, other behavioral changes that may be seen in aging dogs include pain-related irritable aggression and behavioral changes reflective of declines in sensory capabilities.

Research is limited and unclear about the impact of CDS on survival (61–63). However, the clinical perception among veterinarians is that house soiling and other components of CDS are frequently cited by owners as reasons for euthanasia. More research is needed to determine the impact of age-associated behavioral changes in general, not only CDS, on quality of life and mortality risk in aging dogs. Measures of the behavioral manifestations of aging would be a valuable component of the CGS assessment instrument once the spectrum of changes seen and the impact of these on caregiver burden, quality of life, and mortality are better characterized.

#### Metabolic Changes & Biomarker Candidates

Canine and human aging is characterized by changes in metabolism, and deterioration of metabolic health often precedes age-associated disease and disability. Changes in adipose distribution, insulin sensitivity, and lipid metabolism occur in both humans and dogs. These changes are associated with low grade chronic inflammation and a progressively deteriorating state of metabolic health that represents a key risk factor for age-associated disease in humans and likely in dogs as well. While the role of metabolic dysregulation in association with adverse health outcomes in humans has been well-documented, further research is needed to investigate the utility of metabolic markers in predicting morbidity and mortality risk in the dog. This research will also support the identification and validation of specific biomarkers for metabolic health in dogs that can be incorporated in the CGS definition and assessment.

Despite species differences in the manifestation of metabolic disease, upstream metabolic pathways involved in nutrient and energy metabolism have emerged as core mechanisms contributing to aging across taxa (64, 65). As an example, the metabolic changes seen in overnutrition and obesity are associated with shortened lifespan and increased morbidity and mortality in nematodes, flies, rodents, non-human primates, humans, and dogs (35, 66–68). Influencing these pathways through dietary restriction has proven the most robust intervention to increase lifespan and healthspan by delaying disease onset and mortality in all of these species (67, 69–73).

While metabolic dysregulation is linked with age-associated diseases such as cancer, cardiovascular disease, osteoarthritis, kidney disease, and dementia, early signs of metabolic dysfunction often precede clinical disease (74–78). This suggests that measures of metabolic dysfunction are promising biomarker candidates for earlier detection of at-risk individuals and increased opportunity for preventative interventions. Here we outline key age-associated changes in metabolic physiology observed in dogs that might serve as biomarkers in the CGS assessment. We highlight areas that warrant further investigation in understanding the contribution of metabolic dysregulation to age-associated pathology in dogs and opportunities to validate such potential biomarkers.

##### Adipose Tissue & Adipose Compartment Changes

As in humans, total body fat (adiposity) increases with age in dogs independent of differences in body condition and nutritional status (79). Adipose tissue undergoes structural changes with age, including change in abundance, distribution (a shifting from subcutaneous to visceral depots), cellular composition, and endocrine signaling (80).

In humans, the age-associated redistribution of adipose from subcutaneous to visceral depots plays a central role in the development of insulin resistance, metabolic dysfunction, inflammation, and impaired regenerative capacity with age (81). A similar increase in the ratio of visceral to subcutaneous fat with age has been recently documented in dogs (82).

##### Insulin Resistance

The redistribution of adipose in aging dogs is accompanied by functional metabolic changes, including decreased insulin sensitivity, which is a key potential marker of metabolic health in CGS. This is not surprising given that visceral adipose and central obesity is a significant risk factor for insulin resistance in aging humans. The inverse association between increasing adipose tissue and decreasing insulin sensitivity in the dog is well-documented (83, 84). Evidence for a direct causative link between visceral adipose and insulin sensitivity has also been demonstrated in dogs after omentectomy (85). Studies in companion dogs have shown a positive correlation between increasing visceral to subcutaneous fat ratio and fasting insulin levels and a possible association with surrogate indices of insulin sensitivity (86). Further work is needed to assess the validity and utility of such indices in larger samples and trends with age (83, 87, 88).

Insulin sensitivity, as measured by blood glucose clearance, deteriorates with age in both humans and dogs. This has been documented in both cross-sectional (89) as well as longitudinal studies in dogs (67). Although dogs exhibit greater ability to maintain glycemic control, and therefore develop insulin-dependent diabetes mellitus far less commonly than humans, there is substantial evidence that dogs experience hyperinsulinemia in metabolically dysregulated states, such as overweight and obesity (88, 90, 91). Dogs also display increasing basal insulin levels with age (92, 93).

In laboratory dogs, there is extensive literature documenting mechanisms of insulin resistance. Increasing adiposity due to high fat diet, for example, leads to impaired glucose clearance and increased hepatic gluconeogenesis despite hyperinsulinemia (83, 94–97). A 12-year dietary restriction study performed in Labrador Retrievers demonstrated that improved insulin sensitivity (measured by IV glucose tolerance tests) was associated with increased healthspan and lifespan in calorically restricted dogs (67, 93). Dogs with higher insulin sensitivity were at lower risk of dying and needing treatment for chronic disease (93).

A current challenge in incorporating insulin sensitivity as a marker of metabolic health in the CGS assessment is the lack of sufficiently validated tools for assessing insulin sensitivity in companion dogs (87). Due to their simplicity and ease of use, surrogate index approaches based on steady state fasting insulin and glucose levels have been used extensively in human clinical studies (98), and these have also been used as a proxy measurement of insulin resistance in dogs (99–102). However, it is unclear what these surrogate indices represent physiologically when applied to dogs. There is conflicting evidence concerning the correlation of these indices with dynamic measures of glucose tolerance (83, 103), and the model assumptions for these surrogate indices have not been fully evaluated in dogs (98). While using surrogate indices to assess insulin resistance is a potentially powerful approach, further work is needed to determine their reliability and clinical utility in companion dogs.

In addition, further work needs to be done to understand the contribution of decreased insulin sensitivity to age-associated clinical disease. While dogs are not as susceptible to diabetes mellitus as humans, there are many other negative health consequences associated with insulin resistance in humans and animal models, such as cancer, cardiovascular and neurodegenerative disease, osteoarthritis, and kidney disease (74–77, 104). These may also impact aging dogs, and making those connections is key to establishing the utility of this metabolic biomarker and enable earlier intervention to address age-related metabolic dysfunction.

##### Lipid Profiles & Dyslipidemia

Serum levels of triglycerides, cholesterol, lipoproteins, and free fatty acids change with age in dogs (105). A recent lipidomics study in companion dogs found associations between lipid profiles and body weight (a proxy for estimated lifespan in this species) as well as age (106). Multiple studies have also demonstrated that overweight/obese companion dogs exhibit perturbed lipid profiles (88, 107–109). This is relevant as increased age is a risk factor for overweight and obesity in dogs (110, 111).

Dyslipidemia is a hallmark feature of metabolic dysregulation and a functional consequence of the age-related redistribution of subcutaneous to visceral fat in humans; visceral fat is more lipolytically active and contributes to elevated free fatty acids (FFA), while subcutaneous fat has been shown to act as a sink to decrease circulating FFA (112). Central adiposity is linked with increased lipolysis and hepatic exposure to circulating free fatty acids (FFA) in multiple species, including in the dog, and this is hypothesized to produce hepatic insulin resistance (94, 113, 114).

Another pathophysiological consequence of chronically elevated serum FFA seen in humans and preclinical research studies is the ectopic deposition of lipid in non-adipose tissues, including liver, muscle, pancreas and heart. This lipotoxicity leads to induction of cellular stress and inflammation, immune cell infiltration, impaired tissue function, and cellular senescence (81) and is associated with the pathogenesis of several age-related diseases in humans, including sarcopenia, type 2 diabetes mellitus and fatty liver disease, and cardiac disease (115, 116). A study investigating differences in epaxial musculature in a small sample of young and aged Labrador Retrievers found a negative correlation between degree of CT attenuation and age (117). The authors suggested this may be due to greater muscle fat content, based on the correlation between skeletal muscle CT attenuation and skeletal muscle lipid content found in humans (118). However, there remains a paucity of literature directly examining the prevalence of ectopic lipid deposition with age and the contribution of this phenomenon to tissue dysfunction in the dog; this is an area in which further investigation is needed.

##### Altered Adipokine Secretion and Chronic Inflammation

Adipose tissue secretes inflammatory cytokines, such as TNF-alpha and IL-6, as well as adipose-specific factors called adipokines, key signaling peptides that influence metabolic function and the pathophysiology of many obesity-associated diseases (119). Adiponectin, for example, is an anti-inflammatory adipokine that directly promotes insulin sensitivity and has been associated with improved metabolic health outcomes and longevity in humans and rodent models (120).

A recent meta-analysis of 20 canine studies found that serum adiponectin is lower in dogs with poor metabolic health (e.g., obese and overweight) (111). There is limited evidence suggesting adiponectin decreases with age in dogs, paralleling the case in humans (121, 122), but additional studies are needed to confirm this relationship. However, clinical studies have demonstrated preliminary associations between adiponectin and obesity-related cardiac dysfunction and MMVD, and adiponectin is promising as a biomarker for metabolic health and age-associated disease in dogs (123, 124).

Adipokines influence both local and systemic inflammation by activating receptors on immune cells, and alterations of adipokines are associated with metabolic dysregulation and increased inflammatory cytokines (125). The state of aging-associated, low grade systemic inflammation has been labeled “inflammaging,” and this has been shown to be predictive of cardiovascular disease, multimorbidity, and a decline in physical and cognitive function in humans (126).

While dogs experience increased innate immune system activity and decreasing adaptive immune responses with increasing age, as do humans, there has been scant research investigating the role of chronic inflammation as a risk factor for canine age-associated disease (127–129). Preliminary evidence supports a link between cardiac dysfunction and markers of chronic inflammation in dogs (123). An ongoing, longitudinal research project in aging sled dogs is investigating relationships between steady state circulating cytokines and several functional parameters related to general health, physical fitness, and cognition (130). This and future research should help elucidate the links between aging, chronic inflammation, and age-associated disease in dogs. Once these links are established, biomarkers of inflammation may be useful components of the CGS assessment tool.

#### Frailty

Frailty is a critical concept in human geriatric medicine. Though there are a variety of definitions, the consensus is that frailty involves the loss of strength and function, diminished resistance to stressors, and increased risk of disease, disability, and death (8). Frailty, along with the development of specific diseases, is a function of the diminished robustness and resilience due to aging. This relationship with diminished robustness and resilience has been demonstrated in human clinical studies by linking measures of frailty to risk of adverse health outcomes (morbidity or mortality) or time to recover function from an adverse event (such as hospitalization) (131). Frailty in humans is recognized as a manifestation of aging that interacts with other age-related changes to influence functional and health outcomes in geriatric people (132). Limited research has shown that frailty is associated with aging in dogs, and measurement of frailty would likely constitute a major component of the CGS assessment (133).

There are a number of commonly used instruments to detect and quantify frailty in humans. These generally fall into two categories: the frailty phenotype (FP) (134) and the frailty index (FI) (135). The frailty phenotype is based on the presence or absence of specific clinical measurements, such as strength and walking speed, energy and activity level, and weight loss. The frailty index is based on a cumulative deficit model and consists of a list of diseases and clinical or laboratory abnormalities converted into a continuous frailty score.

Both types of instruments have been shown to predict the risk of disability and other negative health outcomes in humans, including death. There is controversy over the best measurement approach and choice of component variables, but there is consensus that frailty assessment should include measures of function (136, 137). Assessment of frailty is used to guide clinical interventions, and there is some evidence that this approach can improve patient function and reduce the risk of negative outcomes (8, 9).

Because dogs age more rapidly but in a similar manner to humans, it is expected that the concept of frailty will be useful in this species for predicting risk and guiding clinical interventions. Components of frailty, such as decreased mobility, sarcopenia, increased disease incidence, increased need for acute or chronic medical care, and others are associated with chronological age in dogs as they are in humans. Preliminary studies have been conducted to validate both a FP approach and a Canine Frailty Index (CFI) for dogs (133, 138).

A key research need in canine geroscience is the further development and validation of a pragmatic instrument for clinical assessment of frailty in primary care practice. Having such an instrument that can signal an increased risk of morbidity and mortality in individual patients and be used to assess the impact of interventions to reduce this risk would greatly enhance the efficacy of preventative healthcare for dogs.

Assessment of frailty is itself a composite measure of the impact of aging on health and function. However, current approaches to measure canine frailty focus only on a subset of the critical domains of CGS; physical function and disease incidence. Incorporating a measure of frailty into diagnosis of the CGS along with other components measuring physical, functional, behavioral and metabolic manifestations of aging not directly assessed by the FP or CFI would result in a more robust measure of the impact of aging on health and quality of life.

#### Quality of Life

Quality of life (QoL) is considered a critical subject for assessment in elderly humans. It incorporates the impact of aging in multiple domains, including physical health and function, cognition and mood, and psychosocial aspects of life. Health-related quality of life (HRQL) is also predictive of mortality in elderly humans (57, 139).

Just as dogs experience physical and functional decline with aging, they likely experience changes in quality of life similar to those seen in elderly humans. Limited assessment of health-related quality of life in dogs has demonstrated that several dimensions of HRQL decline with age (140). Quality of life assessment is a key factor in decision-making about euthanasia for dogs (141–144), so there is clearly a relationship between perceived QoL and mortality risk.

While there are a number of studies that have developed and demonstrated the usefulness of HRQL instruments in dogs (145–151), there is no consensus on the most appropriate way to measure changes in QoL with aging, and there are limited tools available for doing so. Dog owners commonly cite loss of appetite and mobility, perceived pain, and incontinence as key variables (144). However, less quantifiable or observable factors, such as loss of energy and apparent enjoyment of normal activities, may also be of importance. The specific changes most salient to the perception of quality of life as acceptable or unacceptable may also vary with context or individual owner values.

Given that QoL often appears to decline with age and is associated with the risk of euthanasia in dogs, establishing standards and robust instruments for assessment of QoL in aging dogs is a critical research need. Quality of life is a crucial component of the CGS instrument, and improving our understanding of this variable and how to measure it is necessary for the clinical implementation of the CGS framework.

#### Caregiver Burden

The age-related decline in health and function of companion dogs has significant impact on their human caregivers. There are financial and psychological costs to caring for dogs with chronic illness, so the negative consequences of aging on canine health inevitably also affects the wellbeing of dog owners. Measures of caregiver burden show that caring for a pet with chronic or terminal illness is associated with higher levels of stress, anxiety, and depression and with lower quality of life compared with having a healthy pet (3, 4).

The end of life for most dogs is not due to natural disease but to the deliberate choice by owners to request euthanasia (152–154). This directly links the caregiver burden of age-associated disease and dysfunction in dogs to canine longevity patterns. Large and giant breed dogs, for example, are more likely than smaller breeds to die from chronic, age-related musculoskeletal disease (155, 156). This may well be a function of the greater burden caring for large dogs with impaired mobility places on owners, leading to earlier euthanasia.

A better ability to assess and mitigate the effects of aging on health, function, and quality of life in dogs through implementation of the CGS framework will have indirect benefits for humans by reducing the burden of age-associated disease. This, in turn, will further benefit dogs by reducing some of the drivers of euthanasia.

#### Age-Associated Disease

Aging is a key risk factor for many specific diseases in dogs, including most of the leading causes of canine mortality: neoplasia, cardiovascular disease, and degenerative neurologic or musculoskeletal diseases. The molecular and cellular mechanisms of aging that occur in all individuals are the root cause of many diseases that may seem unrelated because they occur in different organ systems or have differing proximate pathophysiologic causes. Aging can be understood as a collection of common root causes of age-related disease with variable, extended latency to clinical manifestation. This results in a heterogeneous pattern of aging phenotypes among individuals influenced by a complex interaction of many factors.

An important component of CGS is the increased incidence of age-associated disease. Despite significant differences between individuals, breeds, and populations in the specific diseases that manifest with increasing age, the overall increased incidence of age-related disease should serve as both a sign of the health effects of age and as a trigger for interventions to prevent, and even reverse, the influence of aging pathways on morbidity. Some measure of this increased risk should be incorporated into any CGS assessment tool.

Accomplishing this will require a more sophisticated and quantitative understanding of the impact of age on the incidence of specific diseases in specific canine populations. Research linking the core mechanisms of aging and the diseases that companion dogs of various breeds experience will strengthen the predictive value of a disease-incidence component to the CGS.

## Discussion

**Figure 1:**
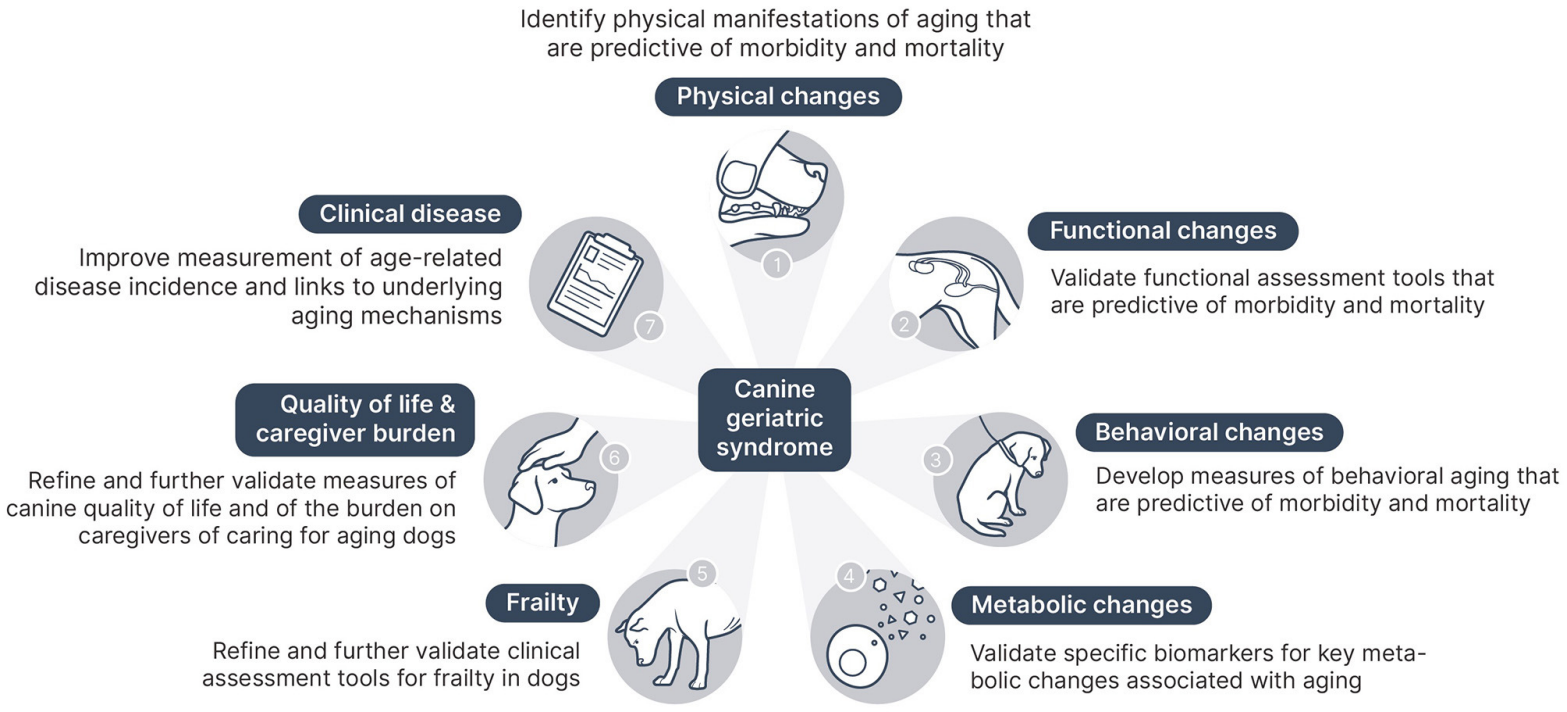
Next steps for developing the components of canine geriatric syndrome.

The purpose of the Canine Geriatric Syndrome framework is to facilitate recognition of the significance of aging as a risk factor for negative health outcomes in dogs and to begin development of a clinical assessment tool for veterinarians to identify and characterize the impact of aging on health and mortality risk in individual canine patients. To achieve this goal, specific components for such a CGS diagnostic tool must be identified and then tested to determine their clinical utility and predictive value. Future research is needed to elucidate the relationships between individual morbidity and mortality risk and the components of the CGS assessment, including specific physical and functional characteristics of aging, laboratory markers of aging pathways and metabolic dysfunction, frailty, quality of life, and caregiver burden. Figure 1 illustrates the key components of a CGS tool and the next steps needed to further develop these.

Once this tool is fully developed and validated, it will have multiple uses. Primarily, the diagnosis of CGS will signal the presence of an age-related increase in the risk of negative health outcomes for individual dogs, which can then trigger preventative and palliative interventions. However, CGS assessment will also be invaluable in evaluating the impact of interventions targeting aging pathways. Improvements in a validated measure of canine aging will be a key goal for studies assessing pharmaceutical, dietary, and other interventions to mitigate or lessen the negative impact of aging on health status.

This article has identified some potential components of the CGS and a CGS assessment instrument as well as knowledge gaps that need to be filled in order to operationalize this framework. We hope this serves as a stimulus for a robust, collaborative effort within the veterinary research community to advance both this framework and the larger field of veterinary geroscience.

## Data Availability Statement

The original contributions presented in the study are included in the article/supplementary material, further inquiries can be directed to the corresponding author.

## Author Contributions

FC developed the core concept of the CGS. BM wrote the first draft of the manuscript. FC and BM wrote sections of the manuscript. All authors contributed to manuscript revision and have read and approved the submitted version.

## Conflict of Interest

BM and FC are employed by Loyal, a biotechnology company developing drug therapies to extend lifespan and healthspan in dogs. The remaining authors declare that the research was conducted in the absence of any commercial or financial relationships that could be construed as a potential conflict of interest.

## Publisher's Note

All claims expressed in this article are solely those of the authors and do not necessarily represent those of their affiliated organizations, or those of the publisher, the editors and the reviewers. Any product that may be evaluated in this article, or claim that may be made by its manufacturer, is not guaranteed or endorsed by the publisher.
